# Cooperators Unite! Assortative linking promotes cooperation particularly for medium sized associations

**DOI:** 10.1186/1471-2148-10-173

**Published:** 2010-06-11

**Authors:** Ádám Kun, Gergely Boza, István Scheuring

**Affiliations:** 1 Evolution and Ecology Program, International Institute for Applied Systems Analysis, Schlossplatz 1, A-2361 Laxenburg, Austria; 2 Department of Plant Taxonomy and Ecology, Institute of Biology, Eötvös University, Pázmány Péter sétány 1/C, H-1117 Budapest, Hungary; 3 Parmenides Center for the Study of Thinking, Kirchplatz 1, D-82049 Munich/Pullach, Germany; 4 Department of Plant Taxonomy and Ecology, Research Group of Ecology and Theoretical Biology, Eötvös University and the Hungarian Academy of Sciences, Pázmány Péter sétány 1/C, H-1117 Budapest, Hungary; 5 Konrad Lorenz Institute, Adolf Lorenz Gasse 2, A-3422 Altenberg, Austria

## Abstract

**Background:**

Evolution of cooperative behaviour is widely studied in different models where interaction is heterogeneous, although static among individuals. However, in nature individuals can often recognize each other and chose, besides to cooperate or not, to preferentially associate with or to avoid certain individuals.

Here we consider a dynamical interaction graph, in contrast to a static one. We propose several rules of rejecting unwanted partners and seeking out new ones, and study the probability of emergence and maintenance of cooperation on these dynamic networks.

**Results:**

Our simulations reveal that cooperation can evolve and be stable in the population if we introduce preferential linking, even if defectors can perform it too. The fixation of cooperation has higher probability than that of on static graphs, and this effect is more prevalent at high benefit to cost ratios. We also find an optimal number of partners, for which the fixation probability of cooperation shows a maximum.

**Conclusions:**

The ability to recognize, seek out or avoid interaction partners based on the outcome of past interactions has an important effect on the emergence of cooperation. Observations about the number of partners in natural cooperating groups are in concordance with the result of our model.

## Background

Evolution of cooperation and altruism remains one of the most intensively studied problems of evolutionary biology [1,2]. On the one hand, the interest is based on the fact that cooperation between competing entities to form a larger, more complex unit played a central role in all the major transitions in evolution [3]. On the other hand, the evolution of altruistic or cooperative acts seems to be a notoriously hard problem, which has provided a challenge for decades. To explain the evolution of cooperation one has to answer the following questions: 1. How can a cooperative (altruistic) act spread in a population where originally only defectors existed? 2. How is the spread of cheaters (agents that enjoy the benefit of cooperation, but don't invest into it) hindered in a population of cooperative individuals?

There are several factors listed which play a central role in the evolution and stability of cooperation [2]. While kin selection is an important mechanism responsible for the evolution of altruistic and cooperative behaviour [4], there are cases when kinship among cooperators or reciprocal altruist is probably too low to explain these behaviours (e.g. [5-15], etc.). The general theoretical framework for studying cooperation of unrelated individuals is the Prisoner's Dilemma (PD) game [16], in which partners can choose either a selfish (defective) or a cooperative strategy. If both partners defect, they get a smaller fitness (*P*) than if both cooperate (*R*), but a defector gets an even higher fitness value when its opponent cooperates (*T*). However, the cooperator receives the smallest fitness of all if its opponent is a defector (*S*). Consequently, although mutual cooperation would result in a higher fitness, defection is the only evolutionarily stable state in this model. Defectors can invade and destroy cooperation in a cooperative population while cooperators cannot spread in a defective population [16,17]. Thus in this situation it remains challenging to explain the emergence and stability of cooperation.

Nowak and May [18] examined a spatial version of the PD game by placing individuals on the nodes of a rectangular grid. Individuals, which can be either defectors or cooperators, can interact only with their nearest neighbours. Payoffs and thus their relative fitnesses are computed according to the PD game. The same individuals are in competition for empty places, and the success of competition is proportional to the relative fitness of an individual. They found that cooperative and defective strategies persist in stable coexistence if the benefit (*b*) of the altruistic act divided by its cost (*c*) ratio is high enough. This polymorph equilibrium of cooperators and defectors is the consequence of limited interaction range among the individuals and limited dispersal as well [19] (but see [20] for an alternative explanation using kin selection).

Grid (regular graph) models are adequate only for sessile organisms or if spatial arrangement of animals also strongly correlates with their associations and/or rank (e.g. [21,22]). However interaction topologies are far from regular graphs in most cases (i.e. individuals don't necessary have the same number of partners, nor are they arranged in the special topology of a regular graph).

For example small world social network structures were found for bottlenose dolphins (*Tursiops sp*.) [23], three-spined stickleback (*Gasterosteus aculeatus*) [24,25] and guppies (*Poecilia reticulata*) [26]. Human social network is also far from a regular graph, as was found for example for an instant messaging network [27], an e-mail network [28] or a scientific collaboration network [29,30].

The study of the evolution and maintenance of cooperative behaviour has recently shifted to employ non-regular graphs. While cooperative strategy survives only if benefit/cost ratio is high for regular graphs [18,31], it dominates for degree heterogeneous graphs (i.e. when individuals can have different number of partners) even if benefit/cost ratio is close to one [32-36]. It is important to note however, that the positive effect of degree heterogeneous graphs on the evolution of cooperation is valid only if the total payoff of an individual is computed as the sum of payoffs received by its neighbours. If payoffs are normalised by the edges then degree heterogeneous graphs behave similar to degree homogeneous graphs (e.g. regular graphs) [37-39]. Similarly, the results are sensitive to the update rule, that is to the rule how local competition, birth and death events are achieved [31]. Finally, cooperators and defectors are present with the same frequency initially in the above mentioned studies, thus these works are focused rather on the stability of cooperative strategies assuming that somehow it become abundant previously, but don't deal with the invasion of cooperators in a population of defectors.

Other recent works focused on the fixation probability of a single cooperator among defectors in different networks [40,41]. Ohtsuki *et al*. [31] have shown that if they use the so called "death-birth" update rule (details see below) then selection favours cooperation (i.e. the average fixation probability of a single cooperator is higher than the fixation probability of a neutral mutant) in the PD game if the *b*/*c *ratio exceeds the average number of neighbours in the network (⟨*k*⟩), that is, if *b/c *> ⟨*k*⟩. They found this relation to be approximately valid in populations of different structure, in which interaction topology is described variously by regular, random regular, random, or scale-free graphs. Taylor *et al*. [41] have proved mathematically that this relation is approximately valid for bi-transitive graphs.

As we emphasized above, the evolutionary stability of cooperation is increased in graph heterogeneous networks [33], while the average probability of invasion of it depends mainly on the average number of neighbours [31,41]. Although this difference seems to be a contradiction, there is a rather simple intuitive explanation of it. If half of the nodes are occupied by cooperators initially then on average half of the hubs are occupied by cooperators as well. These hubs are the core of the spread of cooperators, since if a hub and a sufficient number of its neighbours are occupied by cooperators then its defecting neighbours could not invade this node. If the invasion probability of cooperators is studied then a single cooperator is placed to a randomly selected node which can have different number of edges according to the edge distribution of the network. Fixation probability of a single cooperator increases linearly with edge number of the node (Kun and Scheuring unpublished and [35]), thus the average fixation probability is proportional to the average number of neighbours.

Another feature of the original spatial games is the static nature of the interaction topology. However interaction network are seldom static. Many animals live in fission-fusion societies (African elephants, *Loxodonta africana *[42,43]; bottlenose dolphins, *Tursiops sp*., [44,45]; spotted hyena, *Crocuta crocuta*, [46]; chimpanzee, *Pan sp*., [47]; northern bottlenose whales, *Hyperoodon ampullatus*, [48]; spider monkeys, *Ateles geoffroyi *[49]), where small groups/individuals join and separate iteratively. Besides the above mentioned evidences, associations are non-random in a number of other systems as well (e.g. [25,50-52]). While a simple foraging model can produce non-random association between individuals [53], it is clear that certain animals can very well choose with whom they want to associate. One intriguing example is found in bottlenose dolphins, where individuals can associate with different other individuals for different tasks (foraging, rest, social activity, travel) [54]. This implies that they can form associations based on with whom can they perform a certain act better.

As animals can recognize each other [55,56], there is a possibility to interact only with selected individuals. With respect to cooperation this can have the effect of cooperators preferentially attach to other cooperators, and shy away from defectors. For example, dominant female hyenas offered more food and coalition support to closely associated subordinates, whom in return can help in food capture and defense [7]. Moreover they can discern relative rank [57], thus have the mental capacity to make choices assumed in the presented model.

In the light of field observations, it is becoming increasingly important to study the evolution of cooperation on dynamical networks. Some recent studies focused on the fixation probability of a single cooperator among defectors in the case when graph dynamics is much faster than dynamics of evolution [58,59]. In these works individuals differ in the rate at which they seek a new link. The linking dynamics slightly transforms the payoff matrix, in a way which favours the fixation probability of the cooperative strategy if the life-span of links among cooperators is high enough compared to the life-span of links among cooperators and defectors. In other studies where the relative speed of graph and evolutionary dynamics are varied systematically it is assumed that cooperators and defectors initially are in the same fraction in the population [60,61]. It is found that random relinking is detrimental to the cooperative strategy [62], while preferential link dynamics helps cooperators to prevail in the population [63-67].

Experimental evidences in this field are few and unclear. Croft et al. [26] found that guppies reduce cooperative aid to partners that defected in the recent past, however no such behaviour was detected in another experiment [68].

We know that mere random network dynamic decreases the fixation probability of a single cooperator among defectors, simply because random relinking dilutes the cooperators' associations [69]. This case will serve as a control for our investigation.

**Figure 1:**
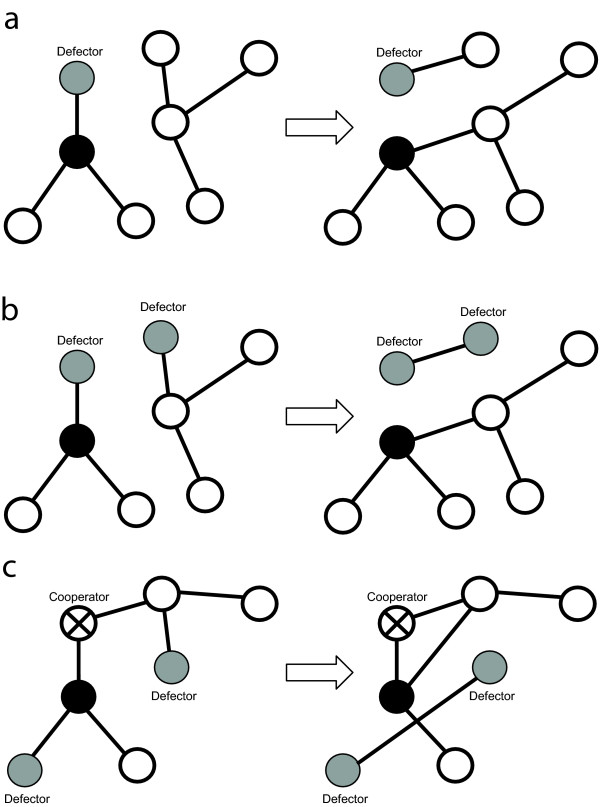
**Three rules for assortative partner choice**. Schematic representation of the different assortative linking. (a) Random choice; (b) Get rid of defectors; (c) The friend of my friend is my friend. Black filled circle represents the focal individual. Gray filled circles represent defectors; circles with a crossed line represent cooperators; and open circle represent an individual with arbitrary strategy.

The aim of this study is twofold. First, we advance our understanding of the evolution of cooperation on dynamical graphs. Here we study how preferential association can affect the fixation probability of a single cooperator among defectors. We compare three different strategies of partner choice/partner rejection: "Random choice" refers to the case when the focal individual chooses to reject a defecting associate, and chooses a new associate randomly. The strategy "Get rid of defectors" allows the new partner to choose which of its associate to reject. The last strategy "The friend of my friend is my friend" is based on triadic closure, which is an important mechanism in the dynamic of social networks [70]. In this case individuals try to associate with associates of trustworthy partners. To study the role of partner choice strategies independently from network topology our defined strategies keep the networks structures intact. (Schematic representation of these strategies are shown on Fig. 1)

Second, we would like to review and synthesize the experimental data available on animal societies with the recent works on games on dynamical graphs. The mainstream literature on evolutionary game theory, while building more and more sophisticated models, has mostly ignored the vast literature on animal behaviour. This is unfortunate as there is a wealth of information available which can make theoretical models more realistic and/or the results more relevant to biology. By comparing and discussing our models in the light of observations and experiments we can in turn hope that empirical researchers will test our and others hypotheses and/or provide us with valuable data for models.

## Results

**Figure 2:**
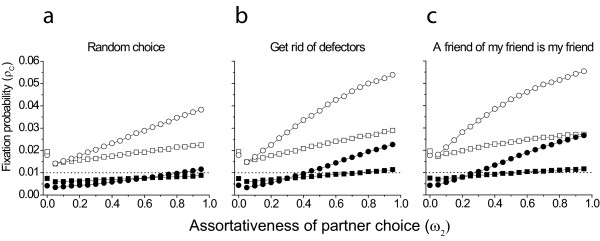
**Fixation probability of cooperators as a function of the assortativeness of partner choice**. In each case cooperation is promoted for some proportion of the ω*_2 _*parameter range compared to random. Squares ⟨*k*⟩ = 4; circles ⟨*k*⟩ = 8. Filled symbols *b *= ⟨*k*⟩, open symbols *b *= 2 ⟨*k*⟩ . *c *= 1, ω*_1 _*= 0.05 and *w *= 0.01 in all cases. The stand alone points represent the case when there is no network dynamics, i.e. ω*_1 _*= ω*_2 _*= 0.0. Dotted horizontal line represents the line above which cooperators fixate with higher than random probability.

Observations show that preferential attachment generally benefits cooperators (Fig 2). The fixation probability of the cooperative strategy increases with higher probability of preferential linking (higher ω*_2_*). As Fig 2 demonstrates cooperative strategy fixates with lower probability when linking is non-selective. Preferential linking can counterbalance it, and the fixation probability can be much higher than observed for a static network. In case of small *b *preferential attachment can increase fixation probability above the referential fixation probability of a neutral mutant in a well mixed population, thus cooperation can spread, where without such preferential attachment it could not (see Fig 2).

Preferential attachment increases the fixation probability of cooperation more if the relative benefit of cooperation (*b*/*c*) is higher or when the mean connectivity ⟨*k*⟩ is higher. Even at lower benefit of cooperation assortative linking can increase the fixation probability to a higher level than expected for a neutral mutant. With high benefit, cooperation would fixate more probably than a neutral mutant even without preferential attachment, but preferential attachment reinforces the evolution of cooperation (compare open vs. solid symbols on Fig 2).

Introduction of more complex relinking rules ("Get rid of defectors" and "The friend of my friend is my friend") (Fig 1b-c) enhances cooperation further. We know from the literature [31], that if the benefit (*b*) can be expressed as *b *= *n*⟨*k*⟩, where *n *is an integer number, then the fixation probability is lower for higher ⟨*k*⟩ at static graphs (at least in the ⟨*k*⟩ = 2-10 range). However, fixation probability increases more with higher ⟨*k*⟩, and surpasses the case with lower ⟨*k*⟩ for dynamical graphs (filled symbols in Fig 2).

**Figure 3:**
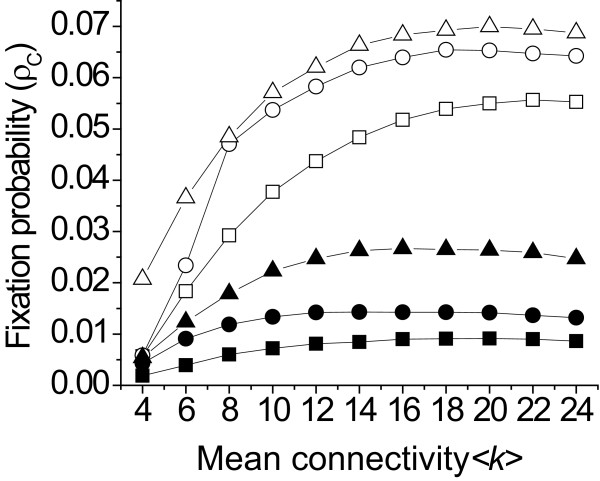
**Fixation probability of cooperation shows an optimum at medium connectedness/association sizes with preferential attachment rules**. Fixation probability of cooperation with different rules for preferential attachment at different average connectivity. Squares represent random relinking; circles "Get rid of defectors"; and triangles "The friend of my friend is my friend". Filled symbols represent *b *= ⟨*k*⟩ and open symbols *b *= 2⟨*k*⟩. *c *= 1, ω*_1 _*= 0.05, ω*_2 _*= 0.95, *w *= 0.01 and in all cases.

Fig 3 shows that there is a ⟨*k*⟩ where the fixation probability is maximal. We have found that this optimal average connectivity is about 14-18.

**Figure 4:**
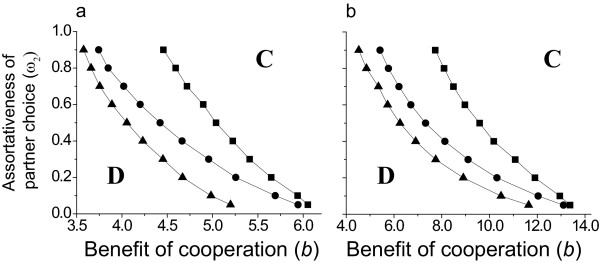
**Areas of the parameter space where cooperation can fixate with higher than random probability**. The areas of cooperator and defector dominance with different relinking rules. Sophisticated relinking rules extend the region where cooperation becomes dominant. The parameter space is shown for the benefit of cooperation (*b*) vs. the assortatitvity (ω*_2_*) **a**. ⟨*k*⟩ = 4, **b**. ⟨*k*⟩ = 8. *c *= 1, ω*_1 _*= 0.05 and *w *= 0.01 in all cases. Squares random relinking; circles "Get rid of defectors"; triangles "The friend of my friend is my friend". A bold letter **D **shows the area where defectors dominate and a bold letter **C **where cooperators dominate.

In the next series of numerical experiments we have interpolated the points where the fixation probability is 1*/N*, the probability of fixation of a neutral mutant in a well-mixed population. Simulations were carried out with ω*_2 _*kept constant, and *b *varied with 0.5 increments (sometimes finer increment is used). The interpolation was done from at least 3 points both above and below the 0.01 probability line (for *N *= 100). The points obtained for the different relinking regimes divide up the parameter space where cooperation can fixate (to the right and up from the point) and where it could not (bottom left corner) (Fig 4).

It can bee seen from Fig 4 that even the simplest random search for other cooperating partners results in a widening parameter space, where cooperation can fixate more effectively than a neutral mutant could. Thus higher probability of preferential linking can lead to cooperation in cases, where otherwise cooperation cannot be attained. Also cooperation can evolve at lower probability of preferential linking for the "Get rid of defectors" and "The friend of my friend is my friend" rules than for the random choice relinking rule. That is, spread of cooperation is possible at even lower values of *b*, if more complex relinking rules are used.

**Figure 5:**
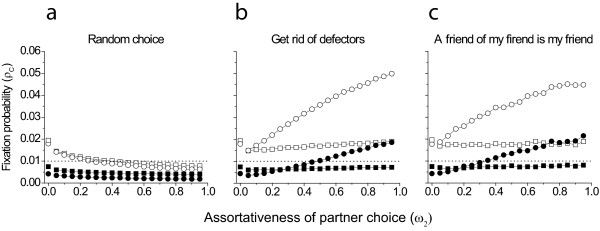
**"Win-stay - loose-relink wisely"**. Fixation probability of cooperators as a function of probability of preferential linking in the PAVLOV scenario. In the PAVLOV scenario, defectors are allowed to seek new partners like cooperators, but preferential relinking still promotes cooperation. In the case of random relinking rule however cooperation is unfavoured. Squares ⟨*k*⟩ = 4; circles ⟨*k*⟩ = 8. Filled symbols *b *= ⟨*k*⟩, open symbols *b *= 2 ⟨*k*⟩ . *c *= 1, ω*_1 _*= 0.05 and *w *= 0.01 in all cases. The stand alone points represent the case when there is no network dynamics, i.e. ω*_1 _*= ω*_2 _*= 0.0. Dotted horizontal line represents the line above which cooperators fixate with higher than random probability.

When we allow defectors to avoid other defectors (PAVLOV scenario) we observe decreasing fixation probability of cooperators as the probability of preferential linking increases (Fig 5). Thus when search for new partner is random, assortative partner choice only promotes cooperation when defectors are not allowed to do the same (compare Fig 5a with Fig 2a). More sophisticated relinking rules however give back qualitatively the same results as above (compare Fig 2b-c with Fig 5b-c), thus even if defectors could avoid their own ilk or try to interact with their cooperative partner's other partners, cooperative behaviour has an increasing probability of fixation with increasing assortativeness. These results also serve as a robustness check for our model, as this assumption does not affect our main results.

## Discussion

We have shown that preferential attachment promotes the evolution of cooperation in graphs where relinking rules were defined to keep the degree distribution constant. A behaviour that allows cooperators to recognize cheaters, and then avoid interaction with such individuals can facilitate the evolution of cooperation in otherwise unfavourable circumstances (Fig 2). This scenario seems to assume that cooperators have higher mental capabilities as only cooperators can recognise exploitation and avoid it (note that cooperators cannot distinguish defectors before interacting with them), but defectors cannot do the same. We have investigated a model setup where both cooperators and defectors were allowed to reconsider their interactions. Except for random choice (Fig 5), the possibility of partner choice increases the fixation probability of cooperators even in this case.

Fixation probability shows a maximum as a function of average connectivity. The intuitive explanation of this result is the following: at low connectivity, a cooperator cluster can easily break up when a defector replaces a cooperator, and dilutes the beneficial effect of cooperator aggregation. However at high connectivity, the invasion of one defector into a cooperator cluster causes only a minor loss to the surrounding cooperators, as they have many other cooperator neighbours. Furthermore, with preferential relinking, the invasion of the defector is generally only temporary as cooperators are working on avoiding it, especially with rule 3 of preferential linking ("The friend of my friend is my friend"). One has a higher chance to find a new cooperator neighbour when one has multiple cooperator neighbours. In contrast, as ⟨*k*⟩ increases, the fixation probability of cooperation decreases [31], because the chance for the emergence of cooperator clusters decreases. So while it is easier to purify cooperator aggregations when the connectivity is higher, the detrimental effect of high ⟨*k*⟩ soon overtakes this advantage. The result of these two effects determines the shape of the fixation probability function.

We note here that the defined relinking rules are so called partner swapping rules which is not very effective against defectors. The consequence of the rule is that a selectively aborted defector definitely has a chance to parasite a new cooperative partner. So our relinking rule is not very effective to oust defectors from the society. However, cooperation associations can form, which associations can be exploited by defectors only temporarily, thus the detrimental effect of defectors on cooperators is only marginal compared to that of solitary cooperators.

While the fact that there is a maximum in the fixation probability as a function of the mean connectivity is intuitive, the value where the maximum appears is not trivial. We found that the fixation probability is maximal at around ⟨*k*⟩. Thus an individual interacts on a long term with around 14-18 other individuals. Interestingly we find rather similar association sizes in cooperative species (Table 1). We do not claim that group size and connectivity are solely affected by the benefit of cooperation. Indeed, group sizes are affected by a great variety of ecological factors. For example, groups of birds benefit from increased vigilance if groups size is larger, as each individual has to be on sentry for less amount of time, but interference competition puts a limit on the size of the group [71]. Another interesting example is that wolves not necessarily infer foraging benefit from group living [72], but larger group can defend the captured prey from scavenging ravens more efficiently [73]. Recently, Voelkl and Kasper [74] have shown that the social network of some 70 primate groups facilitates the spread of cooperation. Thus we find the similarities between the levels of connectivity of real social networks of animals and the optimal connectivity found in our model suggestive.

**Table 1:** Connectivities in animal societies

Species	Connectivity^1^	Ref.
Ring-tailed coatis (*Nasua nasue*)	15.3 ± 6.1	[91]
Ring-tailed coatis (*Nasua nasue*)	17.0 ± 3.2 and 10.2 ± 1.3	[92]
Three-spined stickleback (*Gasterosteus aculeatus*)	14 ± 11	[24]
Three-spined stickleback (*Gasterosteus aculeatus*)	28.2	[25]
Bottlenose dolphins (*Tursiops sp*.)	4.97	[93]
Bottlenose whales (*Hyperoodon ampullatus*)	4.17	[48]
Long-finned pilot whales (*Globicephala melas*)	11-12	[50]
Killer whales (*Orcinus orca*)	2.05	[94]
Spix's disc-winged bat (*Thyroptera tricolor*)	2.9-3.4	[76]
Guppy (*Poecilia reticulata*)	14.7	[26]
Guppy (*Poecilia reticulata*)	14.7; 17.3; 21.9; 22.7	[25]
Grevy's zebra (*Equus grevyi*)	7.08	[82]
Onager (*Equus hemionus*)	10.75	[82]

^1 ^Connectivity was either recorded from an interaction network, or inferred from measurement of association patterns.

We note that long term association or connectivity should not be confused with group (clan, herd, etc.) size. The two can be very different as group sizes can be much larger than presented in Table 1 for connectivity values. For example, observed group size of chimpanzees have a mean of 55, and a maximum of 120 [47]. However, foraging and patrol parties are of size 5-7 [75]. This translates to average connectivity of 4-6 during patrol.

We have assumed that individuals associate for a long time, and while a few changes in partnership within one's life are possible, the network dynamics is comparable to the life cycle of the modeled organisms. Association times are commonly bimodal, as most of the associations last for a short time, and there is some very long-term association (e.g. [50,76,77]). Association between bottlenose dolphins can last for 20 years [44], albeit 7-8 years is more common [23] (they live for about 10-25 years, with maximum of 40-50; [78]). Bottlenose whales usually associate for only a field season, but there is some long term (1-2 years) association between males. Roughly half of the associations of Spix's disc-winged bats last for 150-420 days, and some up to 4 years [76]. One extreme is observed in small, isolated communities, where stable associations only change with birth and death, as observed for a population of Spinner dolphins (*Stenella longirostis*) [79]. One can safely argue, that these long term associations have a larger impact on individual's fitness than the shorter ones, thus our model can capture the relevant time scales for network dynamics observed for certain group living species in nature.

There are some studies where the topology of the interaction (as opposed to links, as here) was allowed to change [80,81].

One possible extension of our model can investigate the effect of multi-tiered social organizations. Social grouping have many levels in a number of species, for example in zebras, where males with harems group for mutual defense [82]; and similar social composition was found in Yunnan snub-nosed monkeys (*Rhinopithecus bieti*) [83]. Furthermore dolphins [44,45] and African elephants [43] also have multi-tiered social organization.

## Conclusions

We have shown that preferential attachment can increase the probability of the fixation of cooperative strategies, and this probability, contrary to what is observed for static interaction networks, is the highest at intermediate level of connectedness. This result is robust independently of how the details of preferential linking are defined and valid in a wide parameter space. The assumptions of our model fits the observations made for real word populations and would explain the higher connectedness of cooperating individuals observed in nature (Table 1). Interestingly the observed group sizes in real word populations appear to be close to the optimal connectedness in our model, where cooperation is easiest to maintain/achieve.

## Methods

Here we considered a population where the interactions are described by a scale-free graph that can vary in time. Scale-free networks were generated according to the method of preferential attachment [84,85]). The population of individuals consisted of defectors and cooperators. An individual derived its payoff, *P *from interactions with adjacent individuals. A cooperator provides help to all individuals to whom it is connected, thus it pays a cost (*c*) for each of its interaction. Neigbours of a cooperator receive the benefit (*b*). Generally, if a cooperator is connected to *k *other individuals and *i *of those are cooperators, then its payoff is *bi *- *ck*. A defector does not provide any help, and therefore its interaction has no costs. However, it still receives the benefit from neighbouring cooperators, thus if a defector is connected to *j *cooperators, then its payoff is *bj*. The fitness of a player *i *is, 1 + *w *+ *wP_i_*, where *w *measures the intensity of the selection and *P_i _*is the payoff of the player. Here we assumed weak selection where the payoff is small compared to the baseline fitness (*w *< < 1).

We employed a "death-birth" updating scheme based on previous studies (e.g. [31,86]), where at each update a randomly chosen individual dies; and subsequently its neighbours compete for the empty site in proportion to their fitnesses. Accordingly, the probability that neighbour *i *occupies the emptied site is  where the fitness of all neighbours are summed. We note here that the success of the cooperative strategy critically depends on the applied update rule and death-birth scheme is the most beneficial update rule for the evolution of cooperation [19,31,86].

The interaction network was allowed to change in the following way. At each update we chose a node randomly. We tested each link of this node, whether they would change or not according to given probabilities, which probability is independent of the past action, and only depends on the composition of the current interaction neighbourhood. If the focal player is a cooperator and a link connects it to a defector, then the probability of changing that link is ω*_2 _*and ω*_1 _*otherwise. Probability ω*_1 _*refers to the basic speed of relinking in the population, assuming that ω*_2 _*> ω*_1 _*means that cooperators selectively shun links with defectors to avoid exploitation, thus ω*_2 _*measures the probability of preferential linking. Individuals have no memory, thus a preferentially aborted connection could be relinked later by chance.

Consequently, we study the spread of a mutant strategy which cooperates and tends to avoid defectors at the same time (ω*_2 _*> ω*_1_*). Thus here we assumed that only cooperators are allowed to avoid defectors with a higher probability. Alternatively we also considered the case when not only cooperators are allowed to avoid defectors with increased probability. In these simulations any individual connecting to a defector had a higher probability of relinking. This strategy is similar to the PAVLOV strategy [87] of "win stay, lose shift".

In order to keep the global degree distribution unchanged, we used a specific rule for relinking. The edge between the focal site and a selected neighbour was exchanged with the edge connecting the chosen site to one of its neighbours. For example, if A to B, and C to D, were connected originally, then after the update, A is connected to C, and B is connected to D. The exchange is allowed only if the new graph remains connected (i.e. there is a path from every node to every other node), and none of the new connections would connect individuals that are already connected. Because the number of edges belonging to a site never changes, the edge distribution of the graphs remain unchanged [88-90].

We investigated three scenarios, which differed in the selection of the edges to be changed, that is different preferential relinking rules.

### (a) Random choice

An edge between the focal site and a randomly chosen site is established. The randomly chosen site loses one of its randomly selected neighbours. This scenario implies that the focal individual searches randomly for a new interaction partner, and the chosen new partner abandons one of its connections at random (Fig 1a).

### (b) Get rid of defectors

An edge between the focal site and a randomly chosen individual is established. Both the focal individual and the randomly chosen individual lose one of their defector neighbours. If an individual does not have a defecting neighbour then a randomly chosen cooperating neighbour is lost (Fig 1b).

### (c) A friend of my friend is my friend

The focal individual tries to establish a link with one of its cooperating neighbour's neighbour. First a cooperating neighbour is selected randomly (or a random individual if none of them cooperates), then one of its neighbours is selected randomly. The focal individual will establish a connection with this individual. The selected new interaction partner loses one of its defecting neighbours, if it has at least one such a neighbour, otherwise connection with a randomly selected neighbour is deleted (Fig 1c). (We note here that the linking update mechanisms used by Santos et al. [61] is somehow similar to our update rule (b) and (c).)

In numerical simulations, we measured the fixation probability of a single cooperator at different levels of graph dynamics (ω*_2_*, ω*_1_*), different average numbers of neighbours (⟨*k*⟩), and variations in the benefit to cost ratio (*b/c*). The initial cooperator was placed in a randomly chosen node, and individuals updated until the whole population consisted of either cooperators or defectors. For each parameter combination we have made 1000 graphs, and on each graph the simulation was repeated 1000 times. The total number of repetitions was thus one million from which we computed the average fixation probability of a single cooperator (*ρ_c_*). Since the fixation probability of a single neutral mutant would be 1/*N*, fixation of cooperators is supported by evolution if *ρ_c _*> 1/*N *[40]). For most of the simulations *N *= 100 was used, except for Fig 3, where *N *= 100. Different population sizes give the same qualitative results.

## Authors' contributions

All authors participated in the design of the study and the writing of the manuscript. ÁK implemented the model. GB and ÁK analyzed the data. All authors read and approved the final manuscript.
